# Improved photovoltaic performance of Pb-free AgBi_2_I_7_ based photovoltaics†

**DOI:** 10.1039/d3na00029j

**Published:** 2023-02-16

**Authors:** Praveen Kumar, Khursheed Ahmad, Shaikh M. Mobin

**Affiliations:** a Department of Chemistry, Indian Institute of Technology Indore Simrol, Khandwa Road Indore 453552 India xray@iiti.ac.in; b Department of Biosciences and Bio-Medical Engineering, Indian Institute of Technology Indore Simrol, Khandwa Road Indore 453552 India; c Center for Advanced Electronics (CAE), Indian Institute of Technology Indore Simrol, Khandwa Road Indore 453552 India

## Abstract

Hybrid perovskites based on bismuth are good candidates for developing lead-free and air-stable photovoltaics, but they have historically been constrained by poor surface morphologies and large band-gap energies. Monovalent silver cations are incorporated into iodobismuthates as part of a novel materials processing method to fabricate improved bismuth-based thin-film photovoltaic absorbers. However, a number of fundamental characteristics prevented them from achieving better efficiency. We examine bismuth iodide perovskite made of silver with improvements in surface morphology and a narrow band gap, and we achieve high power conversion efficiency. AgBi_2_I_7_ perovskite was used in the fabrication of PSCs as a material for light absorption, and its optoelectronic proficiencies were also studied. We reduced the band gap to 1.89 eV and achieved a maximum power conversion efficiency of 0.96% using the solvent engineering approach. Additionally, simulation studies verified an efficiency of 13.26% by using AgBi_2_I_7_ as a light absorber perovskite material.

## Abstract 2

Bismuth based hybrid perovskites light absorbers are good candidates for developing lead-free and air-stable photovoltaics, but they have historically been constrained by poor surface morphologies and large band-gap energies.

## Graphical abstract

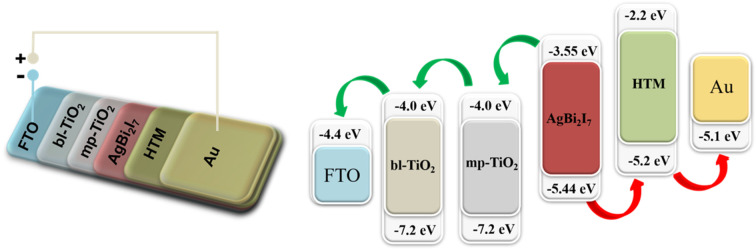

Due to their exceptional semiconducting characteristics, such as relatively low carrier recombination rates,^1,2^ long carrier diffusion lengths,^2^ low charge carrier mobilities,^3,4^ stoichiometry-tunable band gaps,^5,6^ and high absorption coefficients, lead-based halide perovskites have recently attracted a lot of attention.^7^ Formamidinium lead iodide (FAPbI_3_), one of the perovskite systems, has drawn the most interest because of its outstanding performance in thin-film solar cells, where it can achieve a power conversion efficiency (PCE) of 25.7%.^8^ However, the presence of noxious Pb and the product's fragility when exposed to moisture and temperature have led to grave worries about its viability for commercial use. There has been a lot of interest in developing halide perovskite solar cells that are non or low-toxic and air stable. As a result, efforts have been made to develop perovskite solar cells and seek alternatives to lead. Many perovskites based on tin (Sn)^9^ and germanium (Ge)^10^ have been investigated to address the issue of toxicity. Song *et al.*^11^ designed and manufactured Sn-based perovskite materials containing caesium (Cs) as a cation, with CsSnI_3_ and CsSnBr_3_ perovskites achieving efficiencies of 3.04% and 1.83%, respectively. In addition, Ke *et al.*^12^ used a unique hollow 3-D perovskite [enFASnI_3_] as a light absorber material in perovskite solar cells (PSCs), with a 7.1% efficiency. Mhaisalkar and colleagues used a Ge-based AGeI_3_ perovskite-like material.^13^ These Sn and Ge based perovskite-like materials have a high efficiency, but they must be handled with caution since they are air sensitive and need an inert environment to be stable. The perovskite structure is distorted by the quick shift in the oxidation states of Sn and Ge by +2. Moreover, when compared to Pb, these Sn and Ge based perovskites are unable to reach high efficiency. However, instability and poor performance of Sn and Ge based devices under ambient conditions owing to disproportionation are disappointing.

There have been several reports on the use of copper (Cu) as a metal ion in PSCs, utilising a lead-free approach. Mathews *et al.*,^14^ Ahmad *et al.*,^15^ and Li *et al.*^16^ utilized MA_2_CuCl_*x*_Br_4_−_*_x_*_ and C_6_H_4_NH_2_CuBr_2_I perovskite materials as light absorbers in photovoltaic applications. Although these Cu-based perovskites have high stability, they are inefficient in producing good PCE. Yang *et al.*,^17^ Wang *et al.*,^18^ and Vargas *et al.*^19^ studied the optoelectronic activity of (C_6_H_5_CH_2_NH_3_)_2_CuBr_4_, (H_3_NC_6_H_4_NH_3_)CuBr_4_, and Cs_4_CuSb_2_Cl_12_ perovskite materials, respectively. Further, lead(ii), bismuth (Bi^3+^), and antimony (Sb^3+^) ions are isoelectronic (6s^2^), and they may be stable and safe substitutes in thin-film photovoltaic (PV) systems. Bi^3+^ may be used to manufacture PSCs as a non-toxic metal ion, which is promising for replacing Pb and Sn metals. The A_3_Bi_2_I_9_ basic formula (A = Cs^+^, MA^+^, NH_4_^+^, B = Bi^3+^, Sb^3+^, X = Cl^−^, Br^−^, I^−^) has been widely employed in the design and manufacture of lead-free perovskite solar cells. Mobin *et al.*^20^ created Cs_3_Sb_2_I_9_ and Cs_3_Bi_2_I_9_ perovskites with a PCE of above 1%. Hebig *et al.*^21^ and Ahmad *et al.*^22^ used MA_3_Sb_2_I_9_ for photovoltaic applications, whereas Kumar *et al.*^23^ and Zuo *et al.*^24^ used (NH_4_)_3_Sb_2_I_9_ perovskite as the light absorber and developed a device that demonstrated the potential of Bi^3+^ in PSCs. Okano *et al.*^25^ and Ahmad *et al.*^26^ employed a gas-assisted and two-step manufacturing technique to prepare (CH_3_NH_3_)_3_Bi_2_I_9_ for PSCs, respectively. Kulkarni *et al.*^27^ used an *N*-methyl pyrrolidone-assisted method and achieved 0.31% PCE. Huang *et al.*^28^ obtained 0.06% PCE using fluorinated perylene diimide (FPDI) as an ETL (electron transport layer) in the (CH_3_NH_3_)_3_Bi_2_I_9_ PSC. This low PCE might be due to the FPDI's weak surface or charge extraction issue. Sun *et al.*^29^ and Zhuang *et al.*,^30^ on the other hand, looked into the crystalline properties of (NH_4_)_3_Bi_2_I_9_ perovskite and used it in X-rays and PSCs, respectively. Furthermore, their broad band gap and low PCE reduce the likelihood of commercialization. To improve photovoltaic efficiency, 3D structures based on silver-bismuth iodide are used. Filip *et al.* suggested double halide perovskites like Cs_2_BiAgCl_6_ and Cs_2_BiAgBr_6_ in 2016.^31^ AgBi_2_I_7_ perovskite has recently attracted interest because of its efficiency and narrow band gap (<2.0 eV), which improve light harvesting characteristics. Kim *et al.*^32^ reported the first Ag-based PSC using Bi as the metal ion in 2016 and attained a PCE of 1.2%. However, when Shao *et al.*^33,34^ and Johansson *et al.*^35^ employed the same methodology, the performance of AgBi_2_I_7_ perovskite changed with photovoltaic efficiency. Reproducing the solar cell yielded just 0.52 and 0.4% efficiency.^33–35^ AgBi_2_I_7_ perovskite's sensitivity can make up for its lack of repeatability, or it could be caused by the annealing temperature. To comprehend and modify the attributes of Ag-based Bi PSCs, we synthesised AgBi_2_I_7_ (SBI).

We have studied the solution engineering approach to improve the photovoltaic performance of silver-based bismuth iodide perovskites as light absorbers. To our knowledge, this is the first report on the use of SBI perovskites as light absorbers in the presence of DMF and DMF : MeOH. The impact of the solvent engineering strategy on the fabrication of SBI perovskite solar cells may be immediately seen in their efficiency and photovoltaic properties. In the present work, we employed DMF and MeOH, two different solvents in an appropriate ratio, and with molar ratio 1 : 2 of AgI and BiI_3_ respectively. AgBi_2_I_7_ DMF (SBI-D) and AgBi_2_I_7_ DMF : MeOH (SBI-DM) were spin coated onto the conductive glass electrode (FTO) at 1500 rpm for 30 seconds (Scheme 1). Other fabrication data are provided in the ESI.† A convincing demonstration of the equimolar ratio of both the solvent (DMF : MeOH) gives the highest PCE as compared to only 0.96% with DMF under 1 sun illumination conditions and 30–40% humidity.

**Scheme 1:**
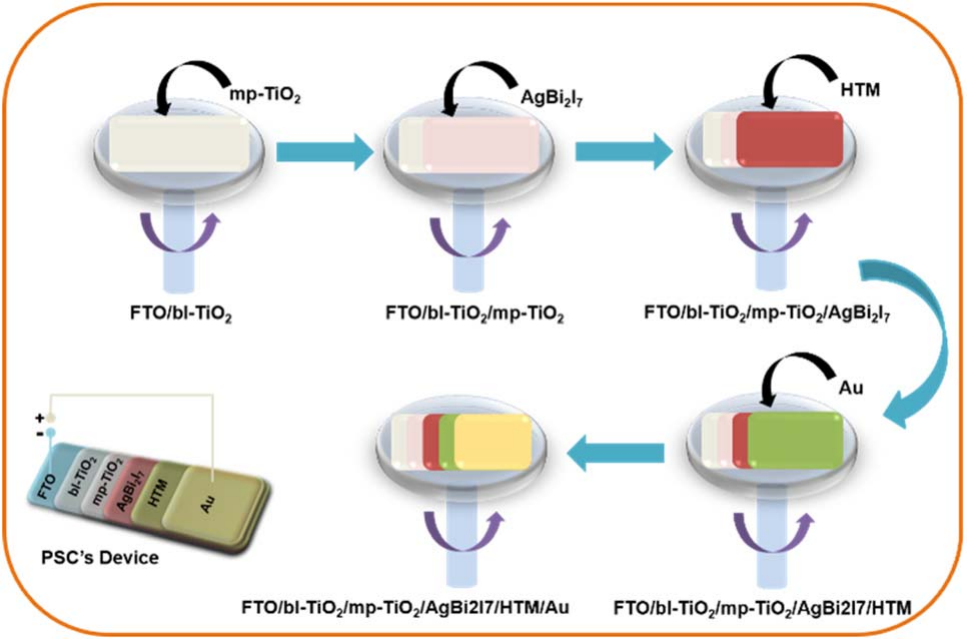
Schematic of the fabrication procedure of the perovskite solar cell.

PXRD was used to characterise the phase purity and formation of the SBI perovskite material synthesized with both DMF and DMF : MeOH solvents, with the findings shown in Fig. 1A. The formation and crystalline nature of SBI-D (DMF : MeOH = 1 : 0) and SBI-DM (DMF : MeOH = 0.5 : 0.5) perovskite materials were revealed by PXRD peak patterns. The growth of SBI-D and SBI-DM is supported by the appearance of a prominent diffraction peak in the (333) plane.

**Fig. 1:**
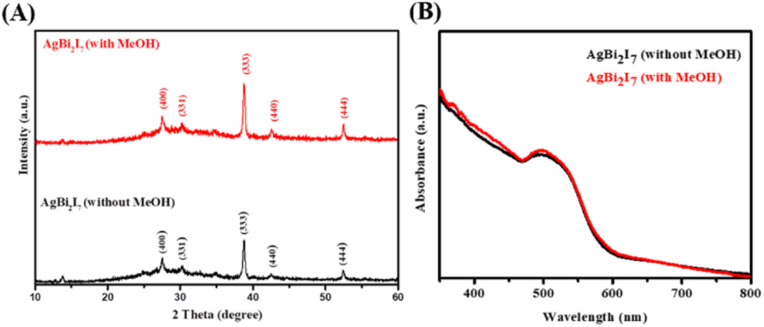
PXRD peak pattern (A) and UV-vis absorption spectra (B) of AgBi_2_I_7_ perovskite with MeOH (red) and without MeOH (black).

The stability of perovskite may be assessed using the Goldschmidt tolerance factor (*t*); when the value of “*t*” is between 0.8 and 1, it implies that the perovskite structure is stable.^36^ Another important component in the cubic crystal is the ionic radius of A, which should not be either large (then, *t* > 1) or too small (then, *t* < 0.8) in comparison to the ionic radius of B. If the ionic radius of A is substantially bigger than that of B, it will not fit inside the BX_8_ octahedron. This could originate from a distinct perovskite structure. The tolerance factor and octahedron ratio are described by eqn (a) and (b):a
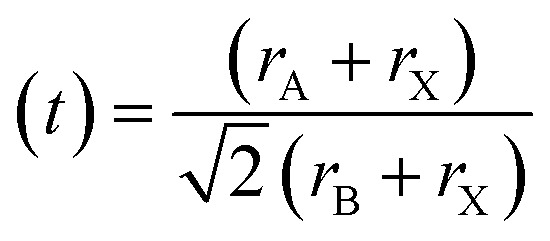
b
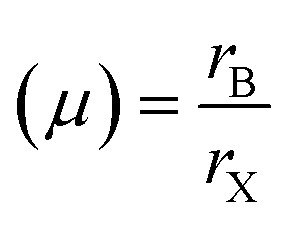
where *r*_A_, *r*_B_, and *r*_X_ stand for ionic radii of A, B, and X present in the perovskite (ABX_3_) structure.

At *t* = 1, the predicted perfect cubic structure was seen. In order to produce a stable octahedron for a cubic cell, the octahedron factor (*μ*) should be between 0.44 and 0.72. The effective ionic radii of silver (Ag) and bismuth (Bi) are 1.26 Å and 1.03 Å, respectively, which are appropriate for the creation of a stable cubic perovskite structure. We may infer and remark on the stability of our perovskite based on this finding, and therefore, it can also be employed as a light absorber material in photovoltaic applications.

UV/vis spectroscopy was used to determine the optical characteristics of SBI-D and SBI-DM thin films (Fig. 1B). The absorption spectra of these perovskite materials were nearly identical. The optical band gaps for SBI-D and SBI-DM may be calculated using UV absorption spectra and linear extrapolation of Tauc plots (Fig. S1†).^37^ The optical band gap was calculated to be 1.89 eV using the Tauc plot ((*αhν*)^*n*^ against *hν*), where *α*, *h* and *ν* are the absorption coefficient, Planck's constant, and excitation frequency respectively. The computed optical band gap reveals that the produced perovskite has high absorbance and has the potential to be used as a light absorber in solar cells.

Surface morphology investigation was done using FE-SEM to evaluate the impact of engagement and solvent content on the perovskite SBI material. The surface morphology of SBI-D and SBI-DM is shown in Fig. 2A–D, and it was obvious that only the DMF solvent produced a rod-like shape, whereas the DMF : MeOH combination produced a distorted rod-like morphology that was converted to a uniform surface morphology.

**Fig. 2:**
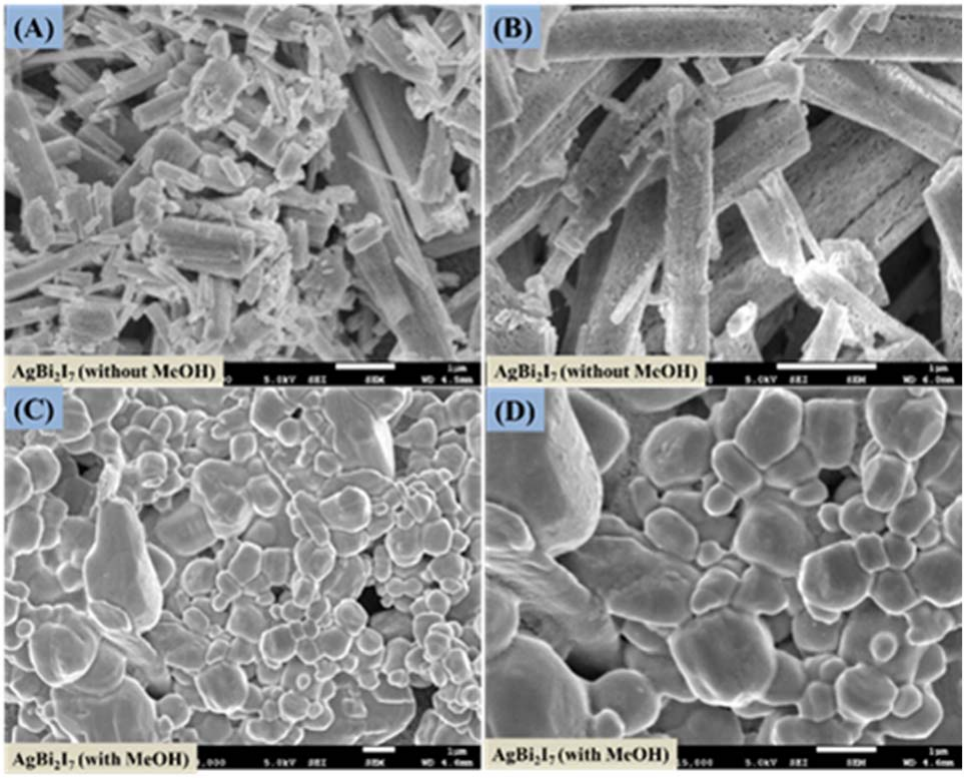
FE-scanning electron microscopy images of AgBi_2_I_7_ perovskite without MeOH (A and B) and with MeOH (C and D).

This might be due to perovskite suppressing and regulating the quick crystallisation process. It has been discovered that a pinhole-free, smooth layer can improve the photovoltaic efficiency of perovskite solar cells.

When a photon strikes a perovskite material, it generates an electron and hole pair. The produced electron was excited and moved towards the LUMO (lowest unoccupied molecular orbital) level, from where it was transferred to conductive glass (FTO) through mesoporous-TiO_2_ and blocking-TiO_2_ LUMO levels. The produced electrons are contained in FTO glass, while the remaining hole in the perovskite material is carried *via* the HTM (hole transport material) to complete the circuit. Scheme 2 explains the entire electron transfer mechanism in simple terms. All of the energy levels of the conductive glass, ETL, and HTM, such as bl-TiO_2_, mp-TiO_2_, Spiro-MeOTAD, and Au, have been extracted from previously published literature. In addition, using UV-visible and cyclic voltammetry (CV) techniques, the HOMO–LUMO energy levels of our perovskite materials SBI were estimated similarly to previous reports.

**Scheme 2:**
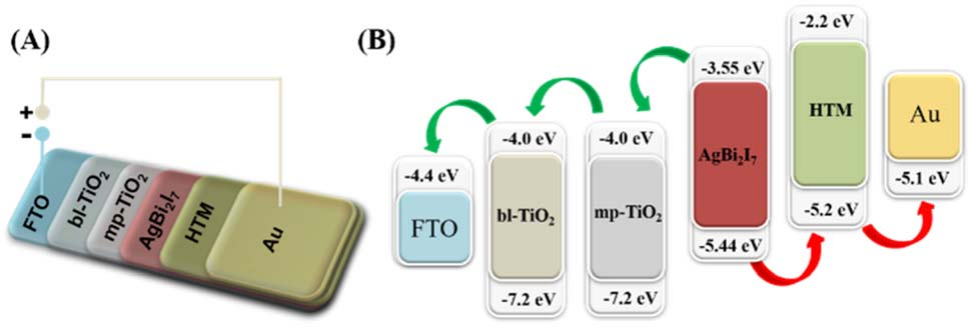
Schematic device structure (A) and materials energy level diagram (B) of the SBI based PSCs. Energy level values as per reported literature.

The optical band gap of SBI was determined using a Tauc plot, whereas the onset reduction potential (*E*_red_) was determined using a CV graph (Fig. S2†). We determine the energy levels of the SBI perovskite material using eqn (i) and (ii).^38^ All of the CV measurement details are included in the ESI.†

i *E*_CB_ (*E*_LUMO_) = −(*E*_red_ + 4.725) eV

ii *E*_VB_ (*E*_HOMO_) = −(*E*_CB_ − *E*_g_) eV

Here, *E*_g_ and *E*_red_ stand for the optical band gap and onset reduction potential, and *E*_CB_ and *E*_VB_ are conduction and valence band energy levels.

Henceforth, we compared our computed SBI perovskite energy levels (*E*_CB_ and *E*_VB_) to the ETL and HTM for smooth charge transfer. The results indicate an excellent correlation between the HOMO and LUMO levels, implying that SBI perovskite has potential as a light absorber material.

Moreover, a PSC device was designed using AgBi_2_I_7_ as a perovskite light absorber; comprehensive device fabrication details are included in the ESI†. The device's photovoltaic performance was measured after it was fabricated under ambient conditions (30–40% humidity). The short circuit photocurrent density–voltage curve was used to assess the device's photovoltaic performance. Fig. 3 shows the photovoltaic performance of SBI-D and SBI-DM under one sun conditions (1.5 AM; 100 mW cm^−2^). Table 1 contains all of the photovoltaic parameters recorded by the devices. The maximum PCE of 0.96% was reached using SBI-DM PSCs, which is higher than the PCE of PSCs manufactured with SBI-D as a light absorber. Additionally, Fig. S5, ESI† provides the box charts of the *J*_sc_, FF, *V*_oc_, and PCE for SBI-D and SBI-DM. Moreover, SBI-DM perovskite as a light absorber attained a higher open circuit voltage than SBI-D PSCs. However, AgBi_2_I_7_ synthesized from DMF and MeOH solutions revealed homogeneous grains and a thin uniform layer, providing an easy interaction with surrounding charge transfer layers. It is observed from the XRD and SEM results that the solvent in the precursor solution significantly affects the crystallization and morphology of AgBi_2_I_7_. This solvent engineering approach showed the improvement in morphology without affecting the perovskite structure, which resulted in an increase in efficiency.

**Fig. 3:**
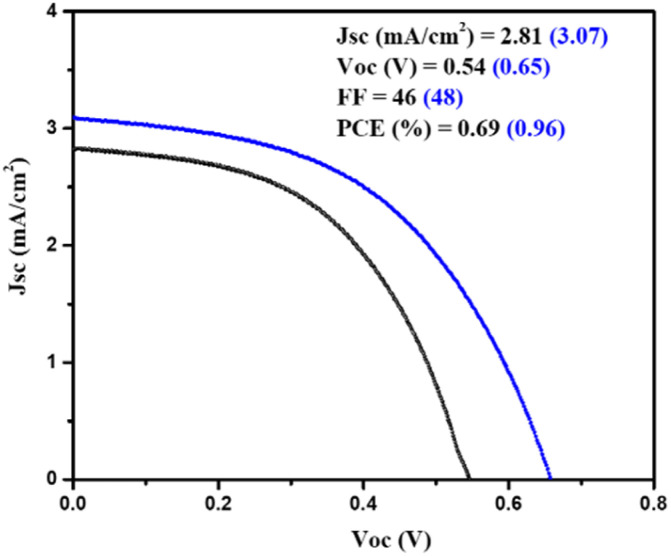
Showing the photovoltaic performance of SBI-D and SBI-DM under 1 sun conditions.

**Table 1:** Showing the comparison of reported photovoltaic performances with SBI-D and SBI-DM

Light absorbers	*V*_oc_ (mV)	FF (%)	*J*_sc_ (mA cm^−2^)	PCE (%)	References
(CH_3_NH_3_)_3_Sb_2_I_9_	896	55	1.0	0.49	21
C_6_H_4_NH_2_CuBr_2_I	200	46	6.2	0.46	16
Cs_2_SnI_6_	520	52	3.2	0.86	39
AgBi_2_I_7_	690	43	2.76	0.83	40
(CH_3_NH_3_)_3_Sb_2_I_9_	740	52	1.48	0.57	41
Cs_2_NaBiI_6_	470	44	1.99	0.42	42
Cs_3_Bi_2_I_9_	570	222	49	0.62	43
(NH_4_)_3_Sb_2_I_9_	1003	115	42.9	0.51	44
CH_3_NH_3_SnBr_3_	490	46	2.2	0.5	45
(MA_3_(Bi_1−*x*_Sn_*x*_)_2_I_9_)	556	48	3.70	0.91	46
**SBI-D**	540	46	2.81	0.69	**This work**
**SBI-DM**	650	48	3.07	0.96	

We summarized all data in Table 1 for comparison with other reported photovoltaic performances of Pb free PSCs. Recently, there has been a tremendous increase in the production of Pb-free PSCs. The research and development of non-toxic perovskite materials for photovoltaic applications has garnered considerable interest from researchers. 0-D (CH_3_NH_3_)_3_Sb_2_I_9_ perovskite was introduced by Hebig *et al.*^21^ as a potential contender for lead-free perovskite solar cells. Additionally, a PCE of 0.49% was obtained by using solvent engineering techniques that included a toluene drop during the spin-coating procedure. Li *et al.*^16^ created a novel form of photovoltaic material with a band gap of 1.64 eV, although they only managed to obtain a 0.46% efficiency. For PSCs, Qiu *et al.*^39^ used a Cs_2_SnI_6_ light absorber, but the manufactured device had a subpar PCE of 0.86%. A thin file of AgBi_2_I_7_ perovskite with a direct band gap of 1.93 eV was designed by Shao *et al.*,^40^ although the efficiency was only 0.83%. 2018 had seen the utilization of the (CH_3_NH_3_)_3_Sb_2_I_9_ perovskite material in solar cells by Chatterjee *et al.*,^41^ and the manufactured PSC device had the best PCE of 0.57% without any dopant. In earlier research, Zhang *et al.*^42^ designed a novel perovskite structure; the light absorber (Cs_2_NaBiI_6_) had good optoelectronic properties, however its PCE was less than 1%. Very stable Pb free PSCs have also been developed using all inorganic perovskite structures, although only 0.62% efficiency was attained.^43^ Zuo *et al.*^44^ had used variation in halide ions using iodide and bromide ions, with (NH_4_)_3_Sb_2_I_9_ having the maximum PCE of 0.5%. However, Yokoyama *et al.*^45^ and Ahmad *et al.*^46^ respectively designed Sn-based perovskites (CH_3_NH_3_SnBr_3_) and Sn-incorporated materials (MA_3_(Bi_1−*x*_Sn_*x*_)_2_I_9_), where the PCE was higher with the doped light absorber (MA_3_(Bi_1−*x*_Sn_*x*_)_2_I_9_). When compared to previous reported lead-free perovskite devices, the photovoltaic performance of our SBI-D and SBI-DM based PSC devices was superior.

Research is still underway to develop a high-performance, stable device that can meet all energy demands. In this viewpoint, various attempts such as encapsulation, insertion of metal ions, doping of metal ions, and multistep fabrication were done. Different Pb-free PSCs have been developed and manufactured. We also looked into the electrical and optical properties of perovskite light absorbers and charge transport layers, which are important for improving the efficiency of perovskite solar cells. Computational studies were conducted to determine the influence of ETL, HTM, and perovskite material thickness during solar cell fabrication. Understanding the variable performance characteristics of PSCs (FF, *V*_oc_, *J*_sc_, PCE) in terms of thickness variation was important. The SCAPS-1D programme was used to simulate the AgBi_2_I_7_ lead-free perovskite.^47^

The *J*–*V* curve and performance metrics for the FTO (500 nm)/TiO_2_ (100 nm)/AgBi_2_I_7_ (varying)/Spiro-MeOTAD (100 nm)/Au device architecture are shown in Fig. 4a and b. According to the numerical simulation, the AgBi_2_I_7_ lead-free perovskite's greatest PCE, with a perovskite layer thickness of 500 nm, was 13.26%. When the perovskite material thickness is extended from 100 nm to 500 nm, the *J*_sc_ value rises while the *V*_oc_ value somewhat declines but not enough to have an impact on performance. The outstanding AgBi_2_I_7_ perovskite PCE of 13.26% at optimum 500 nm thickness was employed for simulation purposes (Fig. 4).

**Fig. 4:**
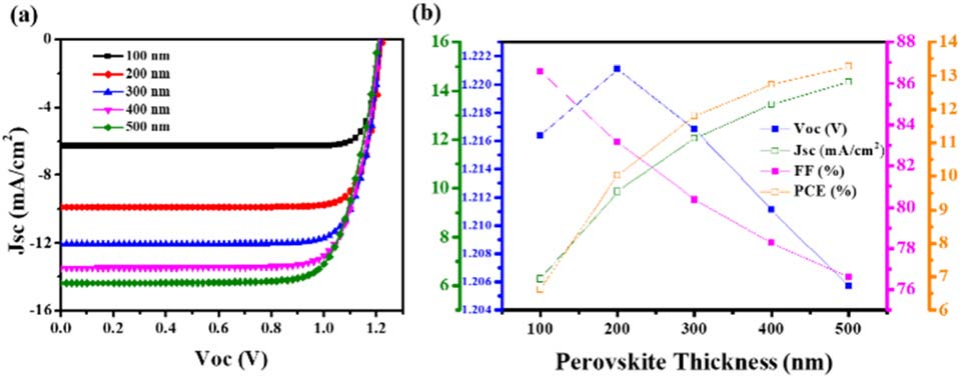
Photovoltaic performance (*J*–*V*) (a) of the simulated Pb-free PSCs with the device architecture of FTO (500 nm)/TiO_2_ (100 nm)/AgBi_2_I_7_ (varying)/Spiro-MeOTAD (100 nm)/Au. Photovoltaic parameters (b) of the simulated Pb-free PSCs with the device architecture of FTO (500 nm)/TiO_2_ (100 nm)/AgBi_2_I_7_ (varying)/Spiro-MeOTAD (100 nm)/Au.

ETL and HTM effects on performance had been examined to note the impact of perovskite material thickness. The *J*–*V* curve of a simulation with increasing TiO_2_ thickness is shown in Fig. S3a.† A little drop in *J*_sc_ was seen when the TiO_2_ thickness increased from 100 nm to 500 nm. Additionally, this drop in *J*_sc_ impacts the device PCE as the TiO_2_ thickness increases, with only a minimal impact on *V*_oc_ and FF (Fig. S3b†). As a consequence, simulation findings show that TiO_2_ at a thickness of 100 nm is far more effective than that of the ETL. Fig. S4a† shows the *J*–*V* curves of AgBi_2_I_7_ perovskite with various thicknesses of HTMs (Spiro-MeOTAD), which exhibited almost little change in *J*_sc_ and *V*_oc_. The simulated results of the perovskite parameter demonstrate that, with the exception of *V*_oc_, all other parameters showed a declining tendency as the thickness of the HTM increased (Fig. S4b†). As a result of its superior performance, modelling of the AgBi_2_I_7_ perovskite was done at a 100 nm TiO_2_ and HTM thickness. Recently, computational studies on lead-free perovskite solar cells were also carried out by Mobin *et al.*^46^ and relative efficiencies of approximately 12% for the perovskite materials MA_3_(Bi_1−*x*_Sn_*x*_)_2_I_9_ were reported.

To summarize our conclusions, we used a solvent engineering technique to fabricate a silver based bismuth perovskite (AgBi_2_I_7_) material as a light absorber for PSCs. The photovoltaic efficiency was improved by combining DMF and MeOH in an optimum ratio. The obtained device performance results demonstrate the efficacy of the solvent engineering method. Likewise, using AgBi_2_I_7_ as a light absorber in the device construction of PSCs resulted in a good PCE (0.96%) and *V*_oc_ (650 mV). In the future perspective, improving system performance may be achieved by comprehending the crystallization process as well as by investigating appropriate charge transport layers and solvents. To improve the optical characteristics of AgBi_2_I_7_, less noxious metals might be inserted or doped into the structure. Additionally, AgBi_2_I_7_ can be applied in different energy-harvesting scenarios.

## Conflicts of interest

There are no conflicts to declare.

## Supplementary Material

NA-005-D3NA00029J-s001
