# Supplementary material for: Improved photovoltaic performance of Pb-free AgBi2I7 based photovoltaics
Source: Nanoscale Adv. 2023 Feb 16;5(6):1624–30. doi: 10.1039/d3na00029j (PMC10012855; doi:10.1039/d3na00029j)
Supplement: NA-005-D3NA00029J-s001 [file NA-005-D3NA00029J-s001.pdf]

Electronic Supplementary Material (ESI) for Nanoscale Advances.
This journal is © The Royal Society of Chemistry 2023

Electronic Supplementary Information

Improved photovoltaic performance of Pb-free $AgBi_2I_7$ based photovoltaics

Praveen Kumar[ǂ], Dr. Khursheed Ahmad[ǂ] and Dr. Shaikh M. Mobin*[ǂ,†,§]

[ǂ]Department of Chemistry, [†]Department of Biosciences and Bio-Medical Engineering and [§]Center for Advanced Electronics (CAE), Indian Institute of Technology Indore, Simrol, Khandwa Road, Indore 453552, India

*Corresponding author: Dr. Shaikh M. Mobin, Email: xray@iiti.ac.in; Tel: +91-7312438752

Table of content

Experimental Section

Characterization Methods

Fig. S1. Tauc plot of $\mathbf{AgBi_2I_7}$

Fig. S2. CV curve (A) and energy level diagram (B) of $\mathbf{AgBi_2I_7}$**.**

Fig. S3. J-V curves (a) of the simulated Pb-free PSCs with device architecture of FTO(500 nm)/TiO_2(varying)/ $\mathbf{AgBi_2I_7}$ (500 nm)/spiro-MeOTAD(100 nm)/Au. Photovoltaic parameters (b) of the simulated Pb-free PSCs with device architecture of FTO(500 nm)/TiO_2(varying)/ $AgBi_2I_7$ (500 nm)/spiro-MeOTAD(100 nm)/Au.

Fig. S4. J-V curves (a) of the simulated Pb-free PSCs with device architecture of FTO(500 nm)/TiO_2(100nm)/ $\mathbf{AgBi_2I_7}$ (500 nm)/spiro-MeOTAD(varying)/Au. Photovoltaic parameters (b) of the simulated Pb-free PSCs with device architecture of FTO(500 nm)/TiO_2(100nm)/ $AgBi_2I_7$ (500 nm)/spiro-MeOTAD(varying)/Au.

Fig. S5. Box charts of Jsc (B), FF (B) Voc (C), and PCE (D) of the SBI-D and SBI-DM

Experimental Section

Materials

We have purchased all the chemicals (BiI_3, AgI, Spiro-MeOTAD, 4-tertbutylpyridine, TiO_2 precursors, bis(trifluoromethylsulfonyl)imide lithium salt, etc.), solvents (chlorobenzene, Methanol and DMF), FTO glass substrates and other precursors from Merck, SRL, Dyesol, Loba, Sigma Aldrich, BAT-SOL, Alfa Aesar and Solaronix. The precursors and chemicals were used without any further purification.

Characterization Methods

The Powder x-ray diffraction (PXRD) investigations were carried out on RINT 2500 V x-ray diffractometer (Rigaku, Japan), (Source=Cu Kα irradiation; λ = 1.5406 Å). Field Emission Scanning Electron microscopic (FE-SEM) images were taken on Supra 55 Zeiss Field Emission Scanning Electron microscope. Optical band gap was calculated using UV-vis absorption spectroscopy on a Varian UV–*vis* spectrophotometer (model: Carry 100). The photocurrent-voltage (*J-V*) curves were recorded under AM 1.5 G condition (100 mW/cm^2 illumination). Cyclic voltammetry (CV) measurements were carried out on Metrohm Potentiostat/Galvanostat using Nova software.

Electrochemical Investigations

The highest occupied molecular orbital (HOMO) and lowest unoccupied molecular orbital (LUMO) energy values of the **$AgBi_2I_7$** were determined by employing a unique UV-vis+CV approach. The CV curve of the **$AgBi_2I_7$** was recorded using a 3 electrode assembly (where glassy carbon electrode acted as working electrode whereas Ag/AgCl and Pt wire electrode worked as reference and counter electrode respectively). The GCE has been used to determine the potential

of the ferrocene/ferrocenium (Fc/Fc^+) in the 3 electrode system. The CV curve of the $\mathbf{AgBi_2I_7}$ was recorded by dissolving $AgBi_2I_7$ in 0.1M tetrabutylammonium hexafuorophosphate ($TBAPF_6$) in acetonitrile having 0.001M ferrocene at scan rate=20mV/s.

Perovskite film preparations

0.7M BiI_3 was dissolved in 1 mL of N,N-dimethylformamide (DMF) using ultra-sonicator for 1h. Further, AgI was dissolved in the resulting solution. The molar ratio of the AgI and BiI_3 was fixed to 1:2. The obtained reaction mixture was filtered through a 0.22 μm PTFE filter. The obtained reaction mixture was denoted as **SBI-D ($\mathbf{AgBi_2I_7}$, DMF)**. For **SBI-DM ($\mathbf{AgBi_2I_7}$, DMF:MeOH),** we have employed two-step deposition method by utilizing solvent engineering approach to prepare the $\mathbf{AgBi_2I_7}$ films. The BiI_3 and AgI were dissolved in the mixture of DMF (0.5mL) and MeOH (0.5mL) and spin coated (1500 rpm 30sec) on to the FTO glass electrode. This prepared film was denoted as **SBI-DM**. The deposited films were annealed at 100°C for 15 min.

Device fabrication

The fluorine-doped tin oxide (FTO) was patterned and cleaned with detergent, water, acetone and 2-propanol using ultra-sonicator for 15 min. The blocking layer of TiO_2 (bl-TiO_2) was deposited using 20 mM titanium diisopropoxide bis(acetylacetonate) solution and annealed at 450°C for 30min. Further a mesoporous film of TiO_2 (mp-TiO_2) was also deposited on to the FTO/bl-TiO_2 and sintered at 500°C for 45 min. Further, the perovskite film (**SBI-D, and SBI-DM**) was deposited on to the FTO/bl-TiO_2/mp-TiO_2 as described above. Further, hole transport material (HTM) layer was deposited on to the FTO/bl-TiO_2/mp-TiO_2/perovskite. The HTM was prepared using spiro-OMeTAD in chlorobenzene (90mg/mL) with bis(trifluoromethylsulfonyl)imide lithium salt (Li-TFSI; 99.95%), tris(2-(1H-pyrazol-1-yl)-4-tert-butylpyridine)-cobalt(III) tris-

(bis(trifluoromethylsulfonyl)imide=FK209) and 4-tert-butylpyridine (4-tBP). The molar ratio of the Li-TFSI, FK209 and 4-tBP was fixed to 0.45, 0.035 and 3.1. Finally Au counter electrode was deposited on to the FTO/bl-TiO_2/mp-TiO_2/perovskite/HTM using thermal evaporation method.

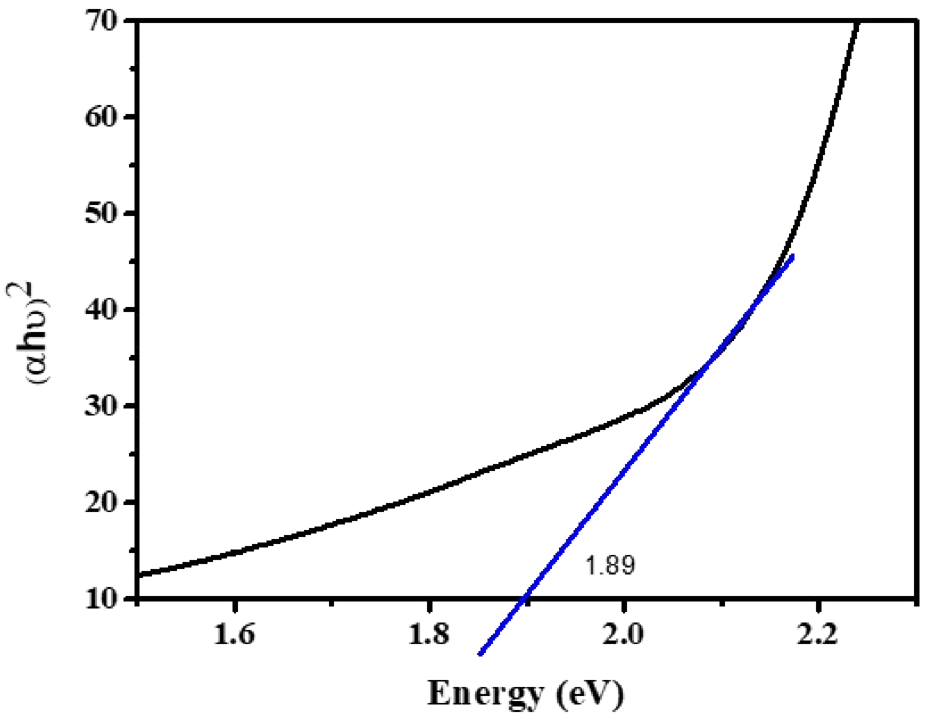

Fig. S1. Tauc Plot of $\mathbf{AgBi_2I_7}$ **.**

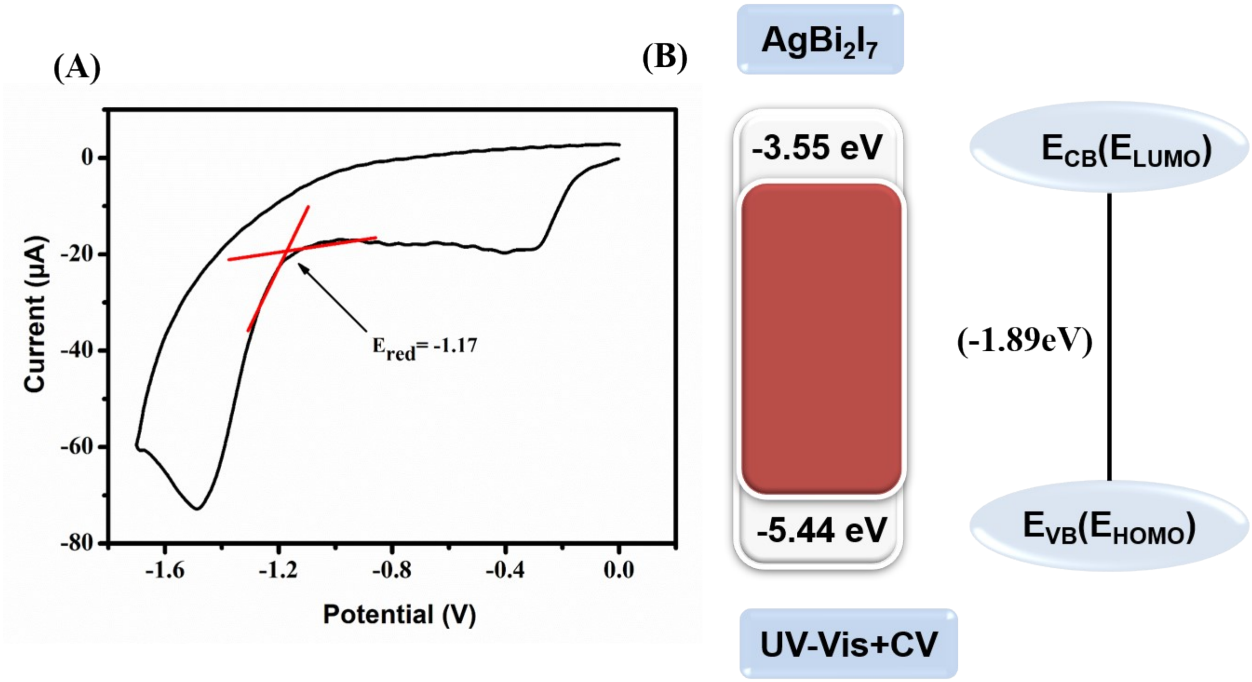

Fig. S2. CV curve (A) and energy level diagram (B) of **$AgBi_2I_7$**.

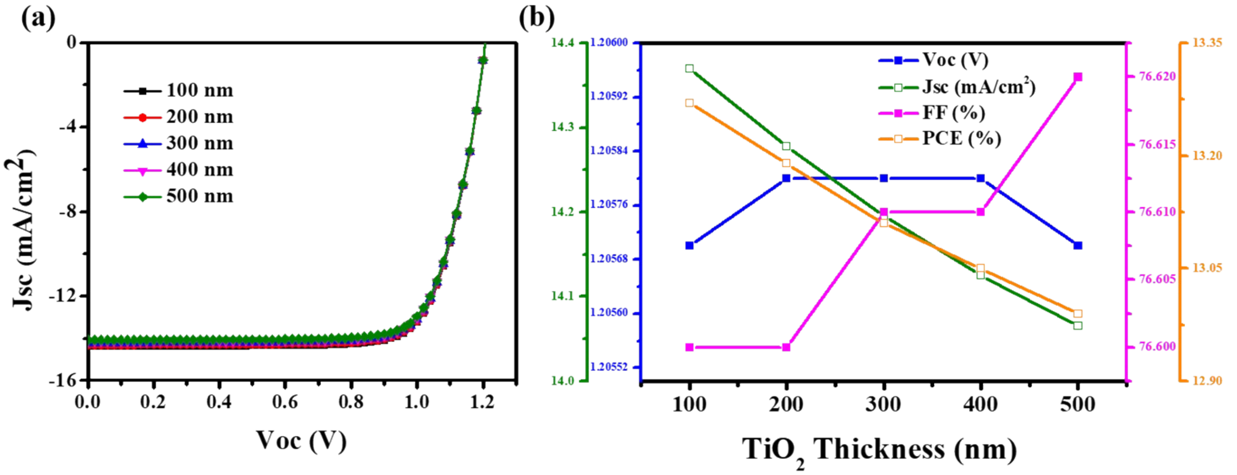

Fig. S3. J-V curves (a) of the simulated Pb-free PSCs with device architecture of FTO(500 nm)/TiO_2(varying)/ $AgBi_2I_7$ (500 nm)/spiro-MeOTAD(100 nm)/Au. Photovoltaic parameters (b) of the simulated Pb-free PSCs with device architecture of FTO(500 nm)/TiO_2(varying)/ $AgBi_2I_7$ (500 nm)/spiro-MeOTAD(100 nm)/Au.

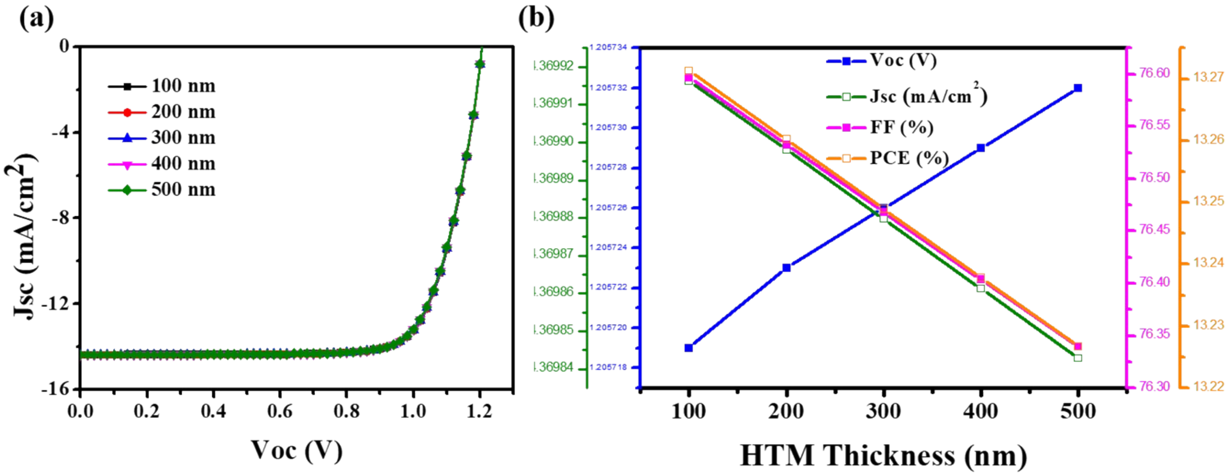

Fig. S4. J-V curves (a) of the simulated Pb-free PSCs with device architecture of FTO(500 nm)/TiO_2(100nm)/ $AgBi_2I_7$ (500 nm)/spiro-MeOTAD(varying)/Au. Photovoltaic parameters (b) of the simulated Pb-free PSCs with device architecture of FTO(500 nm)/TiO_2(100nm)/ $AgBi_2I_7$ (500 nm)/spiro-MeOTAD(varying)/Au.

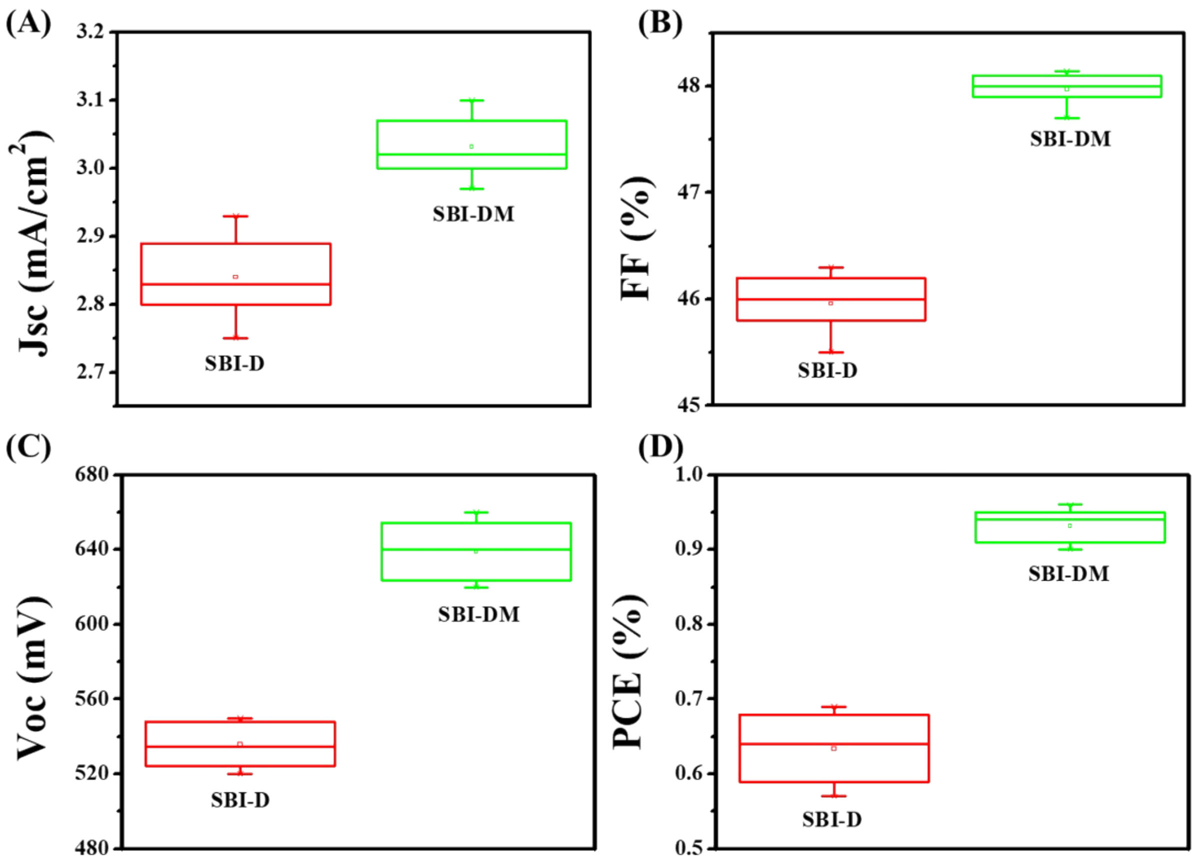

Fig. S5. Box charts of Jsc (B), FF (B) Voc (C), and PCE (D) of the SBI-D and SBI-DM
